# 518. Newborn immune transcriptome changes rapidly and is shaped by maternal SARS-CoV-2 infection and/or vaccination

**DOI:** 10.1093/ofid/ofae631.170

**Published:** 2025-01-29

**Authors:** Zhaohui Xu, Pablo J Sanchez, Shira H Cohen, Leire Pérez Latorre, Traci Pifer, Manish Rijal, Sara Mertz, Rodrigo DeAntonio, Kara Rood, Mahmoud Abdelwahab, Osvaldo Reyes, Anna Bartholomew, Xavier Saez Llorens, Maged M Costantine, Asuncion Mejias, Octavio Ramilo

**Affiliations:** St. Jude Children's Research Hospital, Memphis, TN; Nationwide Children's Hospital - The Ohio State University, Columbus, OH; Nationwide Children's Hospital, Columbus, Ohio; Center for Vaccines & Immunity at Abigail Wexner Research Institute at Nationwide Children's Hospital, Columbus, OH, USA, Madrid, Madrid, Spain; Nationwide Children's Hospital, Columbus, Ohio; Nationwide Children's Hospital, Columbus, Ohio; The Research Institute at Nationwide Children's Hospital, Columbus, Ohio; CEVAXIN Centro de Vacunación e Investigación, Panama, Panama, Panama; The Ohio State University, Columbus, Ohio; West Virginia university, Morgantown, West Virginia; Hospital Santo Tomás, Panama, Panama, Panama; Ohio State University, Columbus, Ohio; Hospital del Niño Dr José Renán Esquivel, Panama, Panama, Panama; The Ohio State University Wexner Medical Center, Columbus, Ohio; St Jude Children's Research Hospital, Memphis, TN; St. Jude Children's Research Hospital, Memphis, TN

## Abstract

**Background:**

SARS-CoV-2 infection during pregnancy is associated with adverse maternal effects, but its impact on infant’s immune development is not well defined. Using transcriptional profiles we analyzed the impact of SARS-CoV2 infection and/or vaccination in pregnant people and their infants longitudinally

Transcriptome changes in pregnant people with SARS-CoV-2 infection and/or vaccination

**Figure:**
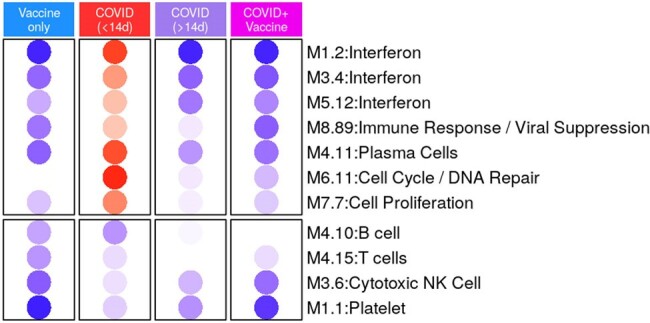

Modular analysis of pregnant people with SARS-CoV-2 infection and/or vaccination compared with healthy uninfected controls. Red dot: overexpression, blue dot: underexpression, white/empty space no difference vs controls

**Methods:**

Multicenter observational study of SARS-CoV-2-infected and/or vaccinated pregnant people and their infants. Pregnant people (infected [n=91], vaccinated [n=42], infected-and-vaccinated [n=14], controls [n=22]) and their infants (n=81, 38,14, 11, respectively) were included. Maternal blood samples were collected during different trimesters and at delivery. Infant blood samples were collected longitudinally from birth (< 72 hours), at 1 week and 1, 3 and 6 months of age. Whole blood RNA was extracted for RNA-sequencing and transcriptomic data analyzed with R

Transcriptional analysis of immune system development in early life

**Figure:**
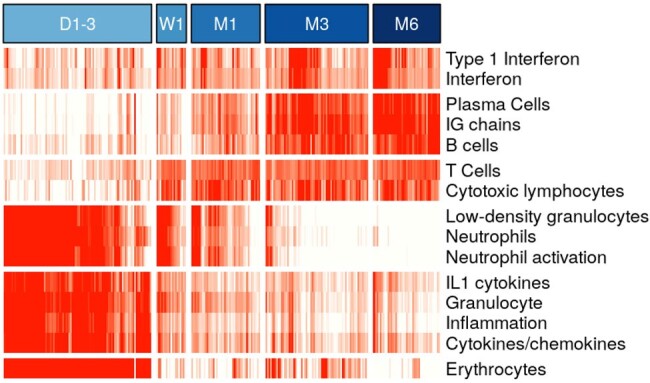

Modular gene expression scores were analyzed longitudinally of individual infant samples. Red: higher expression, white: lower expression. D1-3: days 1 to 3, W1: week 1, M1: month 1, M3: month 3, M6: month 6

**Results:**

Compared with uninfected controls, interferon and plasma cells genes were overexpressed in pregnant people with acute infection (< 14 days), but underexpressed with earlier infection or vaccination during pregnancy (Figure 1). Newborns < 72 h of age (from all groups) showed significantly increased expression of erythrocytes, neutrophils, and inflammation genes. Expression of T/cytotoxic cell genes started to be observed at 1 week, while expression of B cells, plasma cells and interferon genes was observed at 3 months of age (Figure 2). Quantitative gene set enrichment analysis showed that compared to controls, infants from infected and/or vaccinated mothers had increased expression of interferon, while neutrophil, and adaptive immunity genes were decreased (Figure 3)

SARS-CoV-2 infection and/or vaccination of pregnant people shaped immune profiles of their infants

**Figure:**
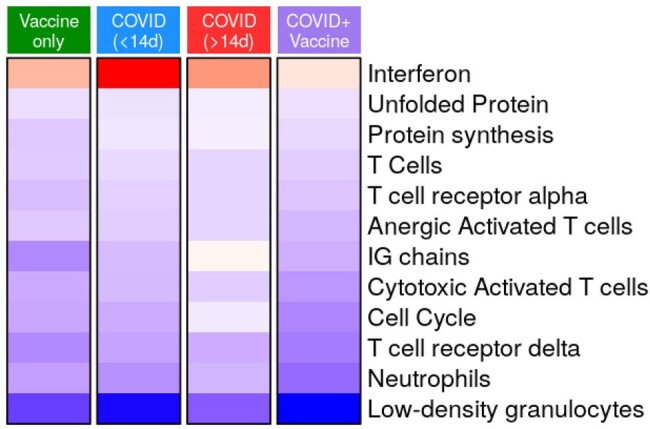

Fold changes of gene sets in infants compared to controls are shown (adjusted p-value <0.01) from quantitative gene set enrichment (QuSAGE) analysis. Red: overexpression, blue: underexpression. Infant groups are defined by maternal infection and/or vaccination status at delivery.

**Conclusion:**

Interferon and plasma cells related pathways were overexpressed in pregnant people with acute COVID-19, while vaccination and SARS-CoV-2 infection earlier in pregnancy were associated with under-expression of both innate and adaptive immunity genes. The infant immune system changed rapidly in the first few days of life, as different immune programs followed separate time trajectories. Maternal SARS-CoV-2 infection and/or vaccination shaped the immune profiles of their newborns, suggesting a potential impact on their immune development

**Disclosures:**

**Rodrigo DeAntonio, MD, MSc, DrPH**, GSK: Grant/Research Support|Moderna: Grant/Research Support|Sanofi: Grant/Research Support **Asuncion Mejias, MD, PhD, MsCS**, Astra-Zeneca: Advisor/Consultant|Astra-Zeneca: Honoraria|Enanta: Advisor/Consultant|Janssen: Advisor/Consultant|Janssen: Grant/Research Support|Merck: Advisor/Consultant|Merck: Grant/Research Support|Moderna: Advisor/Consultant|Pfizer: Advisor/Consultant|Pfizer: Honoraria|Sanofi-Pasteur: Advisor/Consultant|Sanofi-Pasteur: Honoraria **Octavio Ramilo, MD**, Pfizer, Sanofi, Gates Foundation, NIH, and Merck Sharp & Dohme LLC, a subsidiary of Merck & Co., Inc., Rahway, NJ, USA (MSD): Advisor/Consultant|Pfizer, Sanofi, Gates Foundation, NIH, and Merck Sharp & Dohme LLC, a subsidiary of Merck & Co., Inc., Rahway, NJ, USA (MSD): Grant/Research Support|Pfizer, Sanofi, Gates Foundation, NIH, and Merck Sharp & Dohme LLC, a subsidiary of Merck & Co., Inc., Rahway, NJ, USA (MSD): Honoraria|Pfizer, Sanofi, Gates Foundation, NIH, and Merck Sharp & Dohme LLC, a subsidiary of Merck & Co., Inc., Rahway, NJ, USA (MSD): SAC member for MSD

